# Identification of subtelomeric genomic imbalances and breakpoint mapping with quantitative PCR in 296 individuals with congenital defects and/or mental retardation

**DOI:** 10.1186/1755-8166-2-10

**Published:** 2009-03-12

**Authors:** Bernd Auber, Verena Bruemmer, Barbara Zoll, Peter Burfeind, Detlef Boehm, Thomas Liehr, Knut Brockmann, Ekkehard Wilichowski, Loukas Argyriou, Iris Bartels

**Affiliations:** 1 Institute of Human Genetics, Georg August University, Göttingen, Germany; 2 Center for Human Genetics, Freiburg, Germany; 3 Institute of Human Genetics, University of Jena, Jena, Germany; 4 Department of Paediatrics and Paediatric Neurology, Georg August University, Göttingen, Germany; 5 Institute of Human Genetics, University of Lübeck, Lübeck, Germany

## Abstract

**Background:**

Submicroscopic imbalances in the subtelomeric regions of the chromosomes are considered to play an important role in the aetiology of mental retardation (MR). The aim of the study was to evaluate a quantitative PCR (qPCR) protocol established by Boehm et al. (2004) in the clinical routine of subtelomeric testing.

**Results:**

296 patients with MR and a normal karyotype (500–550 bands) were screened for subtelomeric imbalances by using qPCR combined with SYBR green detection. In total, 17 patients (5.8%) with 20 subtelomeric imbalances were identified. Six of the aberrations (2%) were classified as causative for the symptoms, because they occurred either *de novo *in the patients (5 cases) or the aberration were be detected in the patient and an equally affected parent (1 case). The extent of the deletions ranged from 1.8 to approximately 10 Mb, duplications were 1.8 to approximately 5 Mb in size. In 6 patients, the copy number variations (CNVs) were rated as benign polymorphisms, and the clinical relevance of these CNVs remains unclear in 5 patients (1.7%). Therefore, the overall frequency of clinically relevant imbalances ranges between 2% and 3.7% in our cohort.

**Conclusion:**

This study illustrates that the qPCR/SYBR green technique represents a rapid and versatile method for the detection of subtelomeric imbalances and the option to map the breakpoint. Thus, this technique is highly suitable for genotype/phenotype studies in patients with MR/developmental delay and/or congenital defects.

## Background

Mental retardation (MR) is a major health problem affecting 3% of the population[1]. The aetiologies of MR are manifold and include exogenic factors such as prenatal infections, prenatal exposure to teratogenic substances, perinatal asphyxia or genetic factors. However, in up to 60% of the patients the aetiology remains unclear. Chromosomal abnormalities are responsible in 5 to 10% of cases [2]. A substantial proportion of imbalances are microscopically cryptic in routine karyotyping, but can be recognized when analyzing the chromosome ends with more subtle techniques.

Subtelomeric rearrangements are detected in patients with unspecific MR in 3–6% as reviewed by Ledbetter and Martin [3]. In newborns with congenital defects, the detection rate for subtelomeric aberrations is even higher with 9.86% [4]. Therefore, subtelomeric analysis became an important tool in the diagnosis of idiopathic MR, developmental delay (DD) and congenital defects.

Various protocols have been developed to assess subtelomeric aberrations. Most laboratories use fluorescent in-situ hybridisation (FISH) by commercially available BAC probes. Microsatellite polymorphisms were also utilized to detect deletions and duplications [5]. This technique is practically limited by the requirement of parental samples in each case. More recently, multiplex ligation-dependent probe amplification (MLPA) [6] via commercially available kits became widely used. The various diagnostic attempts were reviewed by Rooms et al. [7]. We established a quantitative PCR (qPCR) approach for subtelomeric screening [8]. A probe set of primers for all subtelomeric regions was validated on 20 controls and 20 patients.

Screening of all subtelomeres by PCR based techniques helps to reduce costs when compared to the FISH method, which is the most widely used technique. The first objective of our study was to assess the feasibility of the qPCR approach for subtelomeric screening in routine diagnostics. The second aim was to evaluate whether pre-selection of patients based on family history, pre- and postnatal growth retardation and facial and non-facial dysmorphism as suggested by de Vries et al. [9] can help to improve the predictive value of subtelomeric screening.

Here, we report on a cohort of individuals with normal karyotyping who have MR, DD and/or congenital defects of unknown cause. This is the first clinical application of a qPCR/SYBR green approach for the detection of subtelomeric imbalances.

## Results

Among the 296 patients analyzed, 17 (5.7%) were found to have subtelomeric imbalances. In total, 10 deletions and 10 duplications were identified. Details of the patients with abnormal assay results are given in Table 1. In 5 out of 17 patients, the subtelomeric rearrangement occurred de novo. These aberrations were classified as causative for the phenotype. A deletion or duplication of a subtelomeric region was assessed as a clinically irrelevant polymorphism (copy number polymorphism, CNP) if a parent with a normal phenotype had the same aberration. Six out of 20 subtelomeric rearrangements were classified as CNPs, because they were detected in the patients' phenotypically normal mother or father. One child bears a de novo deletion on chromosome 1q as well as an inherited duplication on chromosome 2p. The mother of patient 6 carries the same deletion on chromosome 6q as her mentally retarded son and is known to display a similar phenotype. Therefore, it can be assumed that this abnormality is also relevant for the phenotype of the patient. In the remaining 4 cases it was not possible to distinguish a causative mutation versus a CNP, because parental DNA samples were not available for analysis.

**Table 1:** Characteristics of patients with subtelomeric aberrations identified by quantitative PCR

**Case No**.	**Age**	**Sex**	**Aberration** **deletion (del)** **duplication (dup)**	**Inheritance**	**Approximate size (Mb, Kb)**	**Clinical Features (in addition to MR/DD)**	**de Vries Score**
1	5 mo	m	del 13q dup 9p	de novo de novo	9.2–11.9 Mb 7.8–8.3 Mb	epicanthic folds; hypertelorism; strabism; microcephaly; small stature, kinking of the aorta; clubfeet; micropenis; cryptorchism; hypotonia; impaired hearing	8

2	1 y 5 mo	f	del 11q dup 6p	de novo de novo	8.5–9.5 Mb 1.8–2.3 Mb	hypertelorism; round face, low set ears; posteriorly rotated ears; microcephaly; small stature; clinodactyly of both 4^th ^fingers; hearing loss; hypotonia; wide spaced nipples; complex heart defect	6

3	10 days	m	del 4p	de novo	1.9–7.7 Mb	growth retardation, microcephaly, prominent glabella, high forehead, prominent nasal bridge, hypertelorism, micrognathia, bilateral single transverse palmar creases, clubfeet	5

4	1 y 11 mo	f	del 1q dup 2p	de novo paternal	5.6–7.1 Mb n.d. (CNP)	microcephaly, small stature, prominent forehead, synostosis of the frontal suture, large earlobes, high palate, cleft uvula, hypoplastic finger and toe nails	4

5	2 y 7 mo	f	dup 19q	de novo	4.8–5.0 Mb	prominent forehead, deep hair line, broad nasal bridge, epicanthus, downward slanting palpebral fissures, low set and posteriorly rotated ears, short neck, midface hypoplasia; dystrophy; pectus carinatum (11)	2

6	5 y 4 mo	m	del 6q	maternal*	4.2–5.3 Mb	high forehead, long philtrum, overfolded helix of both ears, hypotonia	1

7	14 y 3 mo	f	del 1q	n. e. normal sister showed no aberration	> 1.42 Mb	broad, flat nasal bridge; anteverted nares; epicanthus; refractive anomaly; flat philtrum; dental crowding; atrial septal defect; microcephaly; epilepsy; hearing loss; hypotonia; talipes valgus and falt feet; tapering fingers; cortical atrophy; hip dislocation	7

8	5 mo	f	dup 1p	n. e.	> 1.75 Mb	retrogenia; high palate; pterygium colli; broad nasal bridge; anteverted nares; hypertelorism; strabismus; low set ears; overfolded helices; microcephaly; atrial septal defect; urachus cyst; wide distance of mamillas; ventrally positioned anus; hypotonia	5

9	13 y 5 mo	m	del 21q	n. e.	2.4–4.9 Mb	low set ears; broad nasal bridge; anteverted nares; refractive anomaly; retarded bone age; autism; tapering fingers; long toes;	4

10	10 y 9 mo	f	del 20q	n. e.	> 274 Kb	no additional features	0

11	7 y 1 mo	m	dup 10q	n. e.	110–180 Kb	hypertelorism; balcony forehead; hyperopia; hypotonia; hypothyreosis; hearing loss; clinodactyly; retardation of myelination;	5

12	7 y 2 mo	f	dup 10q	paternal	n.d. (CNP)	severe MR; hearing loss; ataxia; seizures; autism;	3

13	9 y 8 mo	m	dup 7p	n. e.	> 792 Kb	epicanthus	0

14	10 mo	m	dup 7p	paternal	n.d. (CNP)	epilepsy; short tapering fingers; clinodactyly; club feet; sandals' gap	6

15	9 y 2 mo	f	dup 7p	maternal	n.d. (CNP)	retardation of speech	0

16	4 y 4 mo	m	del 5p	paternal	n.d. (CNP)	microcephaly; small stature; hypotonia; clinodactyly; plump, big fingers; sandals' gap; pes adductus; pharyngeal cyst; arachnoid cyst; latent hypothyreosis; growth hormone deficiency	5

17	3 y 11 mo	m	del 8p	maternal	n.d. (CNP)	retardation of speech; hemihypertrophy	0

n. e. = not examined; n. d. = not determined; m = male; f = female; MR = mental retardation; DD = developmental delay, y = years, mo = months, *mother also mentally retarded, *de novo *in the mother, CNP = copy number polymorphism

The distribution of the de Vries score [9] in our cohort is shown in Table 2. The median de Vries score of all 296 patients included was 2.55 (range 0–9). Two out of six patients with a causative aberration had a de Vries score of 3 or higher.

**Table 2:** Summary of patients' checklist scores (de Vries et al. [9])

Checklist score	total number of cases	cases with causal aberration	cases with unclear impact of aberration
0	66	1	1

1–2	94	1	0

3–4	80	1	1

5–9	56	3	3

(Polymorphisms are excluded)

### Clinical and molecular findings in patients with confirmed causative subtelomeric aberrations (further details in table 1)

**Figure 1:**
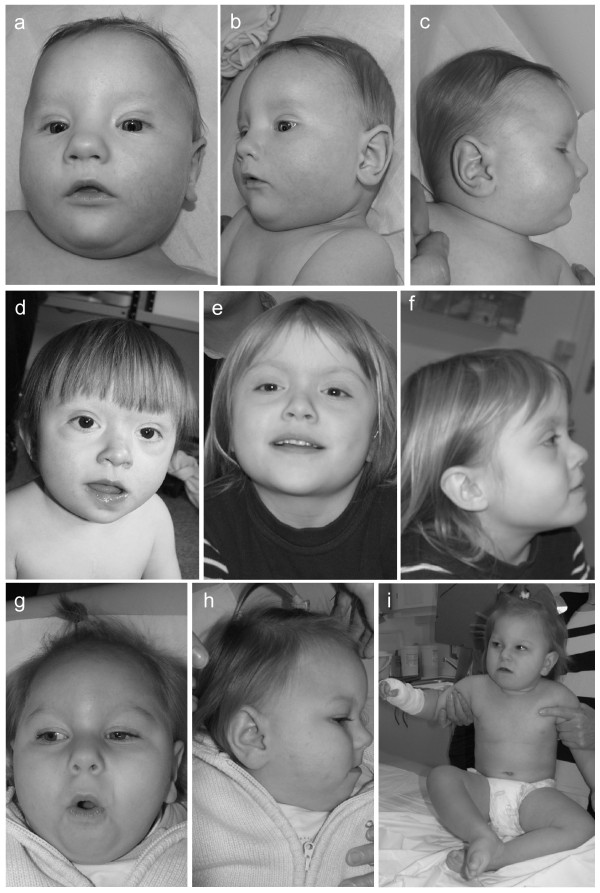
**Photographs of three patients with subtelomeric aberrations**. Patient 1 at the age of 5 months (a-c). Patient 2 at the age of 2 4/12 years (d) and at the age of 5 1/12 years (e, f). Patient 4 at the age of 1 11/12 years (g-i).

Patient 1, with a terminal deletion of 13q (9.2 to 11.9 Mb) and a terminal duplication of 9p (7.8 to 8.3 Mb) is a boy with psychomotor DD. His parents were healthy, and pregnancy was uneventful. He was born small-for-date, body weight, height and occipito-frontal circumference (OFC) stayed below the 3rd percentile. On physical examination at the age of 5 months (height 66 cm, body weight 6340 g, OFC 40.5 cm) he presented with hypertelorism, ptosis, enophthalmus of the left eye, epicanthic folds (see figures 1, a–c), micropenis with cryptorchidism, clubfeet and an overriding 2nd and 4^th ^toe on both feet. The patient was hypotonic but moved all extremities spontaneously, grabbed objects and passed them to the other hand. Hearing was impaired; a cranial magnetic resonance imaging (MRI) showed slightly enlarged ventricles and subarachnoid spaces. Ultrasound examination of the heart revealed a mild kinking of the aorta. An abdominal ultrasound was normal. MLPA confirmed the qPCR results in the patient; FISH analysis of the patient was not performed. Both parents showed normal results in qPCR and FISH analyses, respectively. The duplicated region on chromosome 9p contains at least 68 genes; the deleted region on chromosome 13q encompasses at least 59 genes. To our knowledge, so far no other patient with a similar genotype has been described.

Patient 2, with a terminal 11q deletion (8.5 to 9.5 Mb) and a terminal 6p duplication (1.8 to 2.3 Mb), is a girl with psychomotor DD and a complex heart defect (double outlet right ventricle, pulmonary stenosis, atrial septal defect). She was born small-for-date to healthy parents. On physical examination at the age of 17 months, (height 69 cm, body weight 7800 g OFC 44.5 cm; all below the 3rd percentile) the following facial dysmorphic features were noted: round face, low set and posteriorly rotated ears, depressed nasal bridge, anteverted nares, hypertelorism and an open mouth (see figure 1, d–f)). On both hands, a clinodactyly of the 4^th ^finger was noted, and the patient had wide spaced nipples. The phenotype is reminiscent of Down syndrome. The heart defect was surgically corrected, and a profound bilateral deafness was treated with hearing aids. The patient crawled at 12 months, walked unassisted with 16 months and uttered a few words at 17 months. Testing for a 22q11.2 microdeletion (DiGeorge syndrome) and a mutation screening in the *PTPN11 *gene (Noonan syndrome) revealed no mutation. MLPA confirmed the qPCR results in the patient. Subtelomeric FISH analyses confirmed a deletion of 11qter and revealed an additional 6pter signal on the long arm of the same chromosome 11. These aberrations occurred most likely *de novo *in the patient, because both parents had normal results in qPCR and FISH assays. The duplicated region on chromosome 6p contains at least 16 genes; and haploinsufficiency on chromosome 11q affects at least 275 genes. In many aspects, the phenotype of the patient resembles the 11q terminal deletion disorder (Jacobsen syndrome, OMIM 147791). Surprisingly, thrombocytopenia was not found in our patient, a symptom present in almost all Jacobsen syndrome patients [10].

Patient 3, with a terminal 4p deletion (1.99 to 7.7 Mb) is a boy with profound psychomotor DD. He was born after a 35 weeks of pregnancy with a birth weight of 1490 g, a body height of 43.5 cm and an OFC of 30 cm (all <3rd percentile). Physical examination at age 10 days revealed marked growth retardation, microcephaly, a prominent glabella, high forehead, prominent nasal bridge, hypertelorism, micrognathia, bilateral single transverse palmar creases and clubfeet. The deletion includes at least the Wolf-Hirschhorn syndrome (WHS) critical region. The deletion was confirmed via FISH analysis in the patient; both parents had normal karyotypes and FISH results. Therefore, a *de novo *WHS was diagnosed in the patient. Unfortunately, the exact size of the deletion could not be mapped, because further DNA from the patient was unavailable.

Patient 4, with a terminal 1q deletion (5.64 to 7.14 Mb), is a girl with psychomotor DD. The patient was born to unrelated healthy parents after an uneventful pregnancy at 39 weeks (birth weight 2900 g (25th percentile), OFC 32 cm (3rd percentile)). Developmental milestones were delayed and at the age of 18 months the patient could sit unassisted. At physical examination the patient was 24 months old. She presented as a friendly, mentally retarded child with a body weight of 13 kg (90th percentile), a height of 84 cm (25th percentile) and an OFC of 41.3 cm (2.5 cm below the 3rd percentile). Dysmorphic features comprised microcephaly, prominent forehead, synostosis of the frontal suture, large earlobes, high palate, cleft uvula and a thin upper lip (see figures 1, g–i). Finger and toe nails were hypoplastic. At 23 months of age a cerebral MRI showed globally delayed myelinisation and dysgenesis of the corpus callosum. Testing for FragileX syndrome revealed no abnormality. The terminal 1q deletion was confirmed using both FISH and MLPA analyses. Both parents had normal karyotypes and normal FISH results, respectively. A small duplication of chromosome 2pter was additionally detected in the patient via qPCR. This aberration was also present in the healthy father and therefore considered to be a polymorphism. However, the terminal 1q deletion arose *de novo *in the patient and includes at least 124 genes. Altogether, the patient displays many typical signs, e.g. facial dysmorphisms and brain anomalies that were previously described for other patients with a similar sized deletion of chromosome 1qter [11].

Patient 5, with a terminal duplication of chromosome 19q (4.82 to 5.0 Mb), was previously described in detail [12]. The girl is the second child of healthy, non-consanguineous parents. After a normal pregnancy she was born in the 39th week of gestation with normal body weight, height and OFC. Her developmental milestones were delayed (crawling at 16 months, walking at 2 4/12 years, five words at 2 7/12 years). At the time of investigation she was 2 7/12 years old and presented with a prominent forehead, deep hair line, broad nasal bridge, epicanthus, downward slanting palpebral fissures, low set and posteriorly rotated ears, short neck, midface hypoplasia, poor weight gain (body length and OFC on 25th percentile, body weight on 3rd percentile) and hypotonia. Cranial MRI showed a thin corpus callosum. Testing for metabolic disorders, EEG, echocardiography, abdominal ultrasound, and an X-ray of the left hand was normal. The duplicated region in 19qter contains at least 182 genes. In the child the 19qter duplication could be confirmed with a subtelomeric FISH probe for 19q. An additional signal for 19q subtelomere could be identified on the long arm of chromosome 6. Both parents had normal karyotypes and normal FISH results, respectively.

Patient 6, with a deletion of 6qter (4.2 to 5.3 Mb), was born to a mother with MR. His father was treated for an unspecified psychiatric illness, but was not reported to be mentally retarded or suffering from other symptoms similar to those of his son. The boy was born after a normal pregnancy (birth weight of 3100 g (25th percentile), height of 48 cm (25th percentile), OFC of 35 cm (50th percentile). Developmental milestones were delayed. At the age of 18 months the patient could sit unassisted, he started to walk at 2 years. Speech development was also delayed. During early childhood, the patient had developed absence-like generalized seizures which persisted in low frequency under medication. At the time of investigation, the patient was 6 6/12 years old; body weight was 21.5 kg (50th percentile), body height 116 cm (25th percentile) and OFC 50 cm (3rd – 10th percentile). His mental status was assessed as MR. He presented with a high forehead, long philtrum and an overfolded helix of both ears. The boy was hypotonic and slightly dysmetric in the finger-nose test. An abdominal ultrasound was normal. The deleted region in 6qter contains at least 46 genes. The 6qter deletion was detected by qPCR and confirmed by subtelomeric FISH. The mother of the patient also suffered from a MR, but she showed no further symptoms such as epilepsy. She also had a normal karyotype at 600 bands resolution. However, using qPCR analysis and FISH technique a subtelomeric deletion on chromosome 6q was detected in the mother as well. Both maternal grandparents are healthy and of normal intelligence. The grandparents each had a normal karyotype and no subtelomeric aberrations were detected by qPCR. Thus, the 6qter deletion was maternally inherited in the index patient and occurred *de novo *in his affected mother.

## Discussion

In 5 out of 17 patients in this study the aberration occurred *de novo*; two of them had complex rearrangements with both a duplication and a deletion (patients 1 and 2). As shown by testing the parental samples, none of the abnormalities detected in this study was derived from a parental balanced translocation. Lacking of familial cases is not in line with the observations in large studies [13,14] who assessed that 60% and 50% of pathogenic imbalances, respectively, originated from a parent with a balanced translocation. However, paternity was not checked in our study.

In 5 patients the aberrations detected were inherited from an unaffected parent. These deletions and duplications were rated as familial polymorphisms. Recently, several studies demonstrated that large parts of the human genome and especially the subtelomeric regions are variable in copy number in healthy subject [15,16]. Copy number variations (CNVs) are much more common than previously thought [17]. Our findings also indicate that dosage polymorphisms are frequent and not conclusively determined. Adeyinka et al. [18] reported that one third of the subtelomeric deletions in mentally retarded patients are found in an unaffected parent. But at least in theory, the familial occurrence can not completely exclude a contribution to the phenotype in an affected individual. For example, three of our patients (patients 6, 16 and 17) had familial deletions, which might affect the phenotype by unmasking a heterozygous mutation. Assuming that the maternally derived deletion in patient 6 is indeed pathogenic, the boy expresses a much more severe phenotype than his mother. Variable expression, a phenomenon well known especially in diseases with a dominant mode of inheritance, would be an explanation for the phenotypical differences.

One (patient 12) of two patients with a small dup10q inherited the abnormality from his unaffected father. Recently, duplication of the 10q subtelomere region was described as a common familial variant [14,15], and the size of the 10q imbalance in normal individuals was up to >7.8 Mb [15]. Thus, we propose a benign CNV in one of our other patients (patient 11) with a dup10q despite lacking parental samples.

Three patients carried a duplication of the 7p subtelomere; two of the duplications were inherited from the unaffected mother. To date, the 7p telomere end was not reported to show a copy number variation in large clinical studies (reviewed by Balikova et al. [15]) and the according region is not mentioned as being variable in copy numbers in the Database of Genomic Variants . However, we assume that we detected a new CNV. In general, also familial duplications should be addressed carefully. Rodriguez et al. [19] pointed out that a familial duplication could very well be the cause for affected offspring with apparently the same aberration as the healthy parent. A subtle new rearrangement of the parental duplication during meiosis could result in an altered expression of the relocated genes in the affected child due to a positional effect.

It was not possible to assess the clinical significance in the remaining four cases (patients 8 to 11).

The frequency of MR caused by subtelomeric imbalances in various studies differs widely from 2 to 29% (reviewed by Ravnan et al. [14]). Reasons for this great difference include resolution of prior karyotyping and inclusion criteria. Not all authors excluded the possibility of polymorphisms consistently. Considering large studies, the frequency of truly cryptic subtelomeric abnormalities is estimated to be 2.6% [20] and 2.5% [14]. An overview of previous studies is given in table 3[13,14,20-27]. From the present data we assess that in about half of the 17 patients with an abnormal result the imbalances turned out to be causative for the clinical findings in the patient. The remaining aberrations were rated as benign variants. Therefore, we estimate that the frequency of true causative mutations is approximately 3% in our sample population. In the literature, the most commonly reported clinically significant imbalances were deletions of chromosomes 1p, 22q, 9q, 8p, 2q, and 20p [14]. In our study, after an exclusion of all polymorphisms, imbalances include different aberrations: deletions of 4p, 6q, 11q, 13q and duplications of 2p, 6p, 19p and 19q.

**Table 3:** Surveys estimating the prevalence of significant subtelomere imbalances in individuals with mental retardation and normal cytogenetic results

**Reference**	**Assay used in study**	**Number** **of cases**	**Inclusion/selection criteria**	**No of patients with clinically relevant aberrations**
Vorsanova et al., 1998 (27)	FISH	209	children with MR	8 (3.8%)

Knight et al., 1999 (13)	FISH	466	children and young adults with MR	22 (4.7%)

Riegel et al., 2001 (24)	FISH	254	MR plus dysmorphic signs	13 (5.1%)

Baker at al., 2002 (21)	FISH	250	MR/DD with and without dysmorphisms	9 (3.6%)

Van Karnebeek et al., 2002 (26)	FISH	266	MR	29 (10.9%)

Jalal et al., 2003 (22)	FISH	372	MR with and without dysmorphisms	24 (6.5%)

Yu et al., 2005 (20)	FISH	543	MR with and without dysmorphisms, newborns with malformations/dysmorphisms	7 (1.3%)

Ravnan et al., 2006 (14)	FISH	11,688	MR/DD with a wide range of indications	303 (2.6%)

Koolen et al., 2004 (23)	MLPA	210	MR	7 (4.3%)

Rooms et al., 2006 (25)	MLPA	275	MR	8 (2.9%)

Present study	qPCR	296	MR with and without dysmorphisms	11 (3.7%)

FISH, fluorescence in situ hybridisation; MLPA, multiplex ligation dependent probe amplification; qPCR, quantitative PCR

In 2001, a five-item clinical checklist was suggested [9] as a means of improving the rate of detection of subtelomeric defects among patients with MR. In two thirds of our patients with subtelomeric imbalances, the score suggested by de Vries was ≥ 3 (see table 2). However, even after a clinical reassessment, two patients (patient 5 and 6) had idiopathic MR with only minor features regarding the de Vries score (de Vries score of 2 and 1, respectively). The results of the present study support the assumption that preselecting patients for subtelomeric testing by family history, physical features and growth abnormalities can improve the sensitivity of the screening. Nonetheless, subtelomeric aberrations as a cause for idiopathic MR without associated dysmorphic features are well described [21] and the de Vries checklist score depends on the quality of the clinical observation. Because subtelomeric testing is a relatively uncomplicated diagnostic procedure, it should therefore be considered in patients with unexplained MR.

FISH using commercially available probes was established ten years ago and it is the most widely used technology in subtelomeric screening [28]. However, FISH requires chromosome preparation, is labour intensive and expensive. Polymorphic marker analysis for the detection of subtelomeric imbalances was reported [5]. This technique is automatable, however, the availability of samples of both parents in every case and heterozygosity of a substantial number of loci are indispensable requirements for analysis. Compared to microsatellite analysis, quantitative techniques such as qPCR as well as MLPA do not require parental samples. Quantitative PCR is useful in reducing the workload significantly. It can easily be established in laboratories equipped for molecular genetic diagnostics (ABI System is used for the detection) and is suitable for automated screening. Recently, Udaka et al. [29] developed an assay for the assessment of subtelomeric copy numbers using the primer set by Boehm et al. [8] and detection by multiplex PCR/liquid chromatography. This attempt is of advantage for those laboratories having WAVE technology available, but usually laboratories will not have the ability to perform liquid chromatography. So far no clinical study of a population of mentally retarded individuals has been reported with this method. With commercially available FISH and MLPA kits alone, in most cases it is not possible to address the extension of a detected aberration. In order to study the extension of subtelomeric imbalances, Ballif et al. [30] developed an array CGH approach with a targeted BAC microarray, using probes situated every 0.5 Mb and covering on average 5.7 Mb from the telomere. This strategy allows breakpoint mapping within a range of 1 Mb but requires the establishment of the BAC array technology.

The qPCR approach presented in our study allows the precise determination of the extension of the imbalance. The breakpoint can be narrowed down to less than 1 kb, if primers are selected individually. This could be demonstrated recently in two patients [12,31]. Detailed analysis of the size and breakpoint localisation of the affected region permits genotype-phenotype correlation by combining fine mapping data and clinical features [12]. The results of the present study illustrate the flexibility and feasibility of the qPCR-approach in the genetic evaluation of MR.

The recently released high resolution array comparative genomic hybridisation (CGH) technique [32] offers a whole genome approach to copy number aberrations. This technique has an enormous diagnostic capability. It is obvious that high resolution arrays covering the whole genome would have significant advantages over subtelomeric testing. At that time, array technology requires high priced complex facilities, which are not accessible for all diagnostic centres. In a large clinical study including 1500 mentally retarded children Shaffer et al. [32] confirmed via whole genome array CGH technique that genomic imbalances are frequent (5.6%) in MR. Interestingly, they found that half of the relevant genomic imbalances involved the subtelomeric regions. In principle, those aberrations are detectable by subtelomeric screening as well. As long as genome wide array CGH is significantly more expensive, subtelomeric screening offers a reasonably priced option in the diagnostic work-up of MR. At present, costs for a medium density oligonucleotide array are approximately 300 US$ (Agilent Human Genome CGH 105A array, Agilent Technologies, Inc, Santa Clara, USA), with additional expenses for further reagents, in comparison to approximately 20 US$ reagent costs per qPCR test for all subtelomeric regions.

The effort to clarify initially abnormal results and to distinguish between clinically insignificant CNVs and causative mutations applies to both subtelomeric screening and whole genome array technology in the same extend. CNVs are known to be dispersed over the genome [16]. Some of the polymorphisms are frequent and their detection can be avoided by appropriate assay design. Others are rare in the population and their clinical relevance is unclear, even if they are inherited from a normal parent. Therefore, caution is necessary in the interpretation of the significance of subtelomeric aberrations, especially if parental samples are not available. The present study confirms the diagnostic utility of subtelomeric qPCR with SYBR detection in MR and the versatility of the method in determining the extension of the detected aberrations.

## Conclusion

In conclusion, this is the first report on the clinical application of subtelomeric qPCR with SYBR green detection in patients with idiopathic MR. The results of this study illustrate that this assay represents a rapid and versatile method for the detection of subtelomeric imbalances which implicates the option to map the breakpoint. The qPCR method is also feasible to characterize the precise size of imbalances. Thus, this technique is highly suitable for genotype/phenotype studies in this patient group. Considering the capabilities of whole genome array technologies, the application of the qPCR system is reasonable until microarray analysis is validated and less cost intensive. With a detection rate of up to 3.7% for subtelomeric aberrations in our cohort we could confirm the results of other large studies concerning subtelomeric aberrations in mentally retarded patients [3].

## Subjects and methods

### Patients

In the present study 296 consecutive unrelated patients were included from 2003 until 2007. Patients were collected on the basis of a diagnosis of unspecific MR and/or DD and congenital defects in newborns. Only index cases were included in the database and patients with clinically recognizable syndromes were excluded. The karyotypes of all patients were normal at a 500–550 bands level. At the time of investigation, the patients were 10 days to 49 years of age (median 5.5 years). 4 newborns were included, of which one (patient 3) showed an aberration. 173 patients were male, 123 were female (ratio male/female 1.41). Accessory clinical findings included pre- and postnatal growth abnormalities, facial dysmorphism, non-facial dysmorphism and structural anomalies. Those features and family history were monitored and classified according to the checklist developed by de Vries et al. [9] in order to calculate an individual clinical score for each patient. DNA was extracted from peripheral blood using standard procedures. The study was approved by the ethics board of the University Hospital of Göttingen, Germany.

### Fluorescent Genotyping by Quantitative Real-Time PCR

QPCR with SYBR Green I was established for the detection of subtelomeric imbalances as described [8]. We designed two primer pairs (amplicons) for each subtelomeric region: the first amplicon was constructed inside the genomic sequences from the human FISH-mapped telomere clone set [33] (set A), whereas the other amplicon was mapped relatively to the subtelomeric STS-markers inside the most telomeric clone of the human contig map from the NCBI human databases (set B). Furthermore, we included one amplicon for each q-arm of the acrocentric chromosomes 13, 14, 15, 21, and 22 by selecting a genomic sequence on the q-arm as close to the centromere as possible.

We applied the primer set A to all patients. The second primer set (set B) was used if an aberration was detected with the first primer set. Some of the original amplicons published previously [8] were replaced (see Additional file 1 for further information). Additional primer pairs were designed to address the size of the detected aberrations in patients 1–6. All primers are listed in Additional file 2. For primer design, qPCR conditions and analysis of the obtained data we followed the protocol described by Boehm et al. [8]. All primers were ordered from Operon Biotechnologies GmbH, Cologne, Germany.

The copy number was quantified by the ABI Prism 7900 Sequence Detection System (PE Applied Biosystems, Foster City, CA, USA). Each assay was performed in duplicate and data were analyzed using the Sequence Detection System software (SDS version 2.0, PE Applied Biosystems). Reaction mixtures contained 0.25 mM of each primer and 5 ml QuantiTectt SYBR Green PCR Master Mix (Qiagen Inc., Hilden, Germany). A melting curve analysis of PCR products was performed routinely after finishing amplification.

A standard curve was constructed for each amplicon with serial dilutions of human genomic DNA of an unaffected male individual. Absolute quantification of the amount of the target amplicon was accomplished by measuring its fractional cycle number value and by using the corresponding logarithmic standard curve plot for linear interpolation. Quantitative data were normalized by calculating the Multiple of the Median (MoM) for each amplicon. A MoM value of 1.0 (0.8–1.2) indicated a normal diploid karyotype, and MoM values of 0.5 or 1.5 indicated a monosomy or trisomy of part of the chromosome, respectively. If results were ambiguous, e.g. a MoM value of 1.3, the test was repeated for the specific amplicon.

Whenever possible, aberrations detected by qPCR were confirmed by FISH using subtelomeric probes (Abbott, Des Plaines, IL, USA; Kreatech, Amsterdam, Netherlands) and/or via MLPA using the kits SALSA P036 and P070 human telomere (MRC Holland, Amsterdam, Netherlands) as described elsewhere [34]. If available, parents of probands with subtelomeric imbalances were studied by FISH.

## Abbreviations

MR: mental retardation; qPCR: quantitative PCR; CNV: copy number variation; DD: developmental delay; FISH: Fluorescent in-situ hybridisation; MLPA: multiplex ligation-dependent probe amplification; CNP: copy number polymorphism; OFC: occipito-frontal circumference; MRI: magnetic resonance imaging; *PTPN11: protein-tyrosine phosphatase, nonreceptor-type 11*; WHS: Wolf-Hirschhorn syndrome; EEG: electroencephalography; CGH: comparative genomic hybridisation; MoM: Multiple of the Median.

## Consent

Written informed consent was obtained from the parents of the patients for publication of this case report and accompanying uncensored images. A copy of the written consents is available for review by the Editor-in-Chief of this journal.

## Competing interests

The authors declare that they have no competing interests.

## Authors' contributions

All authors have directly participated in the planning, execution, and analysis of the study and the resulting paper. Specifically, BA and IB were in charge of probe acquisition and project coordination. BA and DB and VB performed qPCR testing, IB and PB performed cytogenetic testing. BA, IB, PB and VB carried out the statistical evaluation. BA, LA, KB, EW and BZ were responsible for patient examination and genetic counselling, TL performed FISH diagnostics. All authors reviewed results, were involved in the preparation of the manuscript and read and approved the final manuscript.

## Supplementary Material

Additional file 1 **Supplementary table 1: Primer details (set A and B).** Primer details for set A and set B primers. Click here for file

Additional file 2 **Supplementary table 2: Primers used for breakpoint characterization. **Details for primers used for breakpoint charakterization. Click here for file
