# Supplementary material for: Identification of subtelomeric genomic imbalances and breakpoint mapping with quantitative PCR in 296 individuals with congenital defects and/or mental retardation
Source: Mol Cytogenet. 2009 Mar 12;2:10. doi: 10.1186/1755-8166-2-10 (PMC2660352; doi:10.1186/1755-8166-2-10)
Supplement: Additional file 1 — Supplementary table 1: Primer details (set A and B). Primer details for set A and set B primers. [file 1755-8166-2-10-S1.doc]

## Supplementary table 1: Primer details (set A and B)

| **Name** | **Primer sequence 5’→3’** | | **Position** |
| --- | --- | --- | --- |
|  | *forward* | *reverse* |  |
| **Set A** | | | |
| 1p008 | TGCAGTTCCTCCGCTGGCCTCCACG | GGGTTTGCAGCTCTGCGCCTGGCAC | 888.104 |
| 1q2 | AATCACAGCCCCACCCACACCTTCC | GCCATCTTGGGTACGGTGGTGCAGA | 246.433.047 |
| 2pFISH | AGGAGGGGTCACCCTCACGCAGTGC | CATCCCTGCCTCAGCCTGTCCCCCA | 151.502 |
| 2q2EUR | TGCGCCCAGACGCCAGCTCATAAA | TCCACAAGCGCCACCATCACCACA | 240.988.816 |
| 3pFISH | TCCTCCGGCCCGTGGTTTTTCGCAG | TGTGAGTGTGCGTGGGGAGGCAGCA | 214.575 |
| 3q500 | CTCCCCAGCTCCCAGCCAGCCTGTAA | TGTCCCATCGCCCCTCCTCTCTGCC | 196.443.993 |
| 4p | tgtccagcgactcatcgcacgtctc | agaaagccgacagccgcagaaggaa | 739.120 |
| 4q | AACCCCAGAGCAGAGCCACAGGAGA | GCTCCATGCGCCAGACACTGTCACT | 190.667.319 |
| 5pFISH | GCCCCCCACACTCCCACCACAGTCA | CGCCCAGCACTCCACCTGCATCCA | 2.219.508 |
| 5q4 | GGGCACCAACGGCAAACGCTATCAG | AGGAAAGACACGCTCGCCCAGGAAG | 180.594.144 |
| 6pFISH | GGGCCTGGACAGAGCCAACTGGGGA | GAGACACAGGGTGGGTGGGGGTGGA | 171.059 |
| 6qFISH | CACAGCACCAGCCCCCTCCCCATCA | TGCCCATCGTGAGGAGCAGGTGCCA | 170.689.569 |
| 7p290* | GGTGCTGAAGGTGCAGAATTCCTCT | CTGGAGTACAGCTCGTACCGGTTGAT | 290.647 |
| 7q | gcacaagagagtgcgccgagcagaa | tgccaggctccacagggacacttga | 158.496.996 |
| 8p | aggtgactttccaggacgctgctgc | tgactgactgccctctgtgctccca | 321.044 |
| 8q | cagccatgccctactcacaccggaa | ggcggcccagaaagcctgaagtaga | 146.041.999 |
| 9pFISH553 | TGGCATGGGAAGGGGATGAGGCCGA | GGCAAATCCCGGTGGAACCCCGACC | 340.993 |
| 9q | ATGCAAGCCGGGCTGGAGACATGA | TCCTTGGCCTCTCACACCGCCATTC | 138.274.701 |
| 10p | GCAGCAGCAAAGAGGCAGCACTGGA | TCTGCCGCCCACTAATCGTTCCCCA | 313.658 |
| 10q20 | GTTGGTCGCAGGTGCCAGGCAGGA | GCAGCAGCCGAGGTGAGCACTGGAA | 135.104.878 |
| 11p | TTAAGGAGCACTGGACGCCCAGCA | GGGTTTCGGTTTCATGCGGCCCAAG | 262.298 |
| 11q | GCCGACAGACACCCACGAAAGGGAA | GAGCATGAGCTGCCACAGGTCCACA | 133.802.622 |
| 12p | CCAGAGGGTCAACATTCGCACCCCA | GGCCCCAGCTTCCTCAACCATAGCA | 92.048 |
| 12q | ACATCCAGGGGCCGTGAACATGAGG | AAGACGCATCACTGGCAGGGACAGG | 131.756.416 |
| 13p | ACTCTGTTGCGAGGGCTCCTCCACA | TACAGGCCAGCTCCAGGCTCAACCA | 20.624.506 |
| 13q* | TCAGGAGGTGATGCTTGGGACGCCA | AGGCAAAAGCAGGCTGTGGGGACAC | 114.012.377 |
| 14p2 | CAGCCCCCAGTGGCCTGAGAGAAAA | GCGCCCATTAGAGACCTGCACCAGA | 20.195.836 |
| 14qFISH250 | AGGAGAACGGGTGGAAAGGAGCCCCTGA | CGCACCCAGCCGATCCAGTAGCTGGTAA | 106.105.955 |
| 15p205* | CCTGTCCATGTTTGAGGTCATCCTG | GTGAAACTCCACACACTCGTCCACAT | 20.550.836 |
| 15q | TTCTGCTCAGCCTCCTCTTGCAGCC | TGTTCCAGCCTCTGCACCTGCTCAC | 99.971.680 |
| 16pFISH | ATCGTCCCCCAGCCCTCCCTCCACA | GCCGAGCACCACCTGCCTTCCCAAC | 57.047 |
| 16qFISH250 | TGGGAGGATCGCAGAGCCCGCAGA | tgctcttcgcgcatccccatcagcc | 88.468.436 |
| 17p265 | CGACCCTGGTCCCAGGCCCATCAGA | GCCCCGGCCTTATCCTCCCAGTCCA | 1.145.788 |
| 17q | TGGGCAGCCCTCATTATCTGGGGCA | ATCCACCCGCCATTGGCATCGAAGC | 78.587.921 |
| 18pEUR | CAGCTTCAATCCGCACAGCCCTCCA | GGGGTTTGGCAAGGGAAGGCAAGGA | 263.912 |
| 18q | TTTGTGGTCTGGGGAGCGGTGAGTG | TGATCCTGCCGGTCGTACACACAGC | 76.042.372 |
| 19p | AGTGCAGCCAGCCACTATACCTGCC | TTCCAAACCATCCACCCCCTCAGCC | 244.803 |
| 19qFISH250 | GCGCCGGAAGACGCCAGAGGAGCTA | ACAGCAACCTGCCCGACCACCCAC | 63.757.194 |
| 20p2 | GGCATCCAGCTCCCACGCAGTCATA | CATGGCACCAGCAGGGAAGGGATTC | 78.177 |
| 20qFISH2 | ATACCCCAGACCCCGCCCCACCAAC | TGGGCAGGAGGTGCAGCCCAGACA | 62.161.784 |
| 21p | TCCCACTCTAGCCTTCCCTGCATGG | GCCACAGGGCAGTCTGTCCATGAGA | 14.613.956 |
| 21qFISH100 | AGGGACCCTCCCCTCAACCGTCCCA | CGCCCCTGCATGGCAGCGTCTGAAA | 46.809.192 |
| 22p16.0* | ACCTGCTCACCTCCCCACTGCTTCCC | ACCTGCCCACTCCCAACTCTCCCCAC | 16.047.836 |
| 22qFISH50 | TGCCCCTGCTTTCCCAGAACCCGGA | TTCCCGCCGACGATGCGGACACC | 49.524.429 |
| Xp2 | CCTCCTATCTGGCACCCCTCCCGCA | GCACAGGCTCGGCTTGGCTGTTTGC | 3.920.513 |
| Xq* | CAATCCTGTGGCAGCAGTGGTG | TCGGCAGGAAGACCAAAGAGGGGAA | 154.167.292 |
| YpEUR | cctgcctcccattgtcaattggtgc | ccggctgcagaggtaatgaagctgg | 7.031.294 |
|  |  |  |  |
| **Set B** | | | |
| 1pFISH | CCCCACCCCATGAGCTGGCACCAGA | CACGCTGCCACCTCGCCTGTCCATC | 1.030.272 |
| 1q | TGGGTGCATGGGTGCTGACATGGTG | AGGCTCCAGGCTGCCTTGCAAACAC | 245.845.108 |
| 2p963* | AGGGAGCAGCGTGAATGGTGGCAGGA | CAGGGGAAGGTGAGGCAGAGGACTGGG | 963.035 |
| 2q269 | GGGCATTGTTGCCCCTCCTTGCCCA | ACACAGCAGAGGCCCAGGGCTCCA | 240.638.788 |
| 3p1200 | CCAGGGCTCCCCACTCACCGTCTGA | GGCTGGGCAGTGGAAGCTGAGCCA | 707.445 |
| 3q2 | CACAAACGCATCCTTCCTGCTGGGC | ACCTGGGCAGAGGCTGTTTCCCATC | 198.555.073 |
| 4qFISH | CCCCAGCAGGCTAACCAGGCTCCCTC | TCGTGGCTCCCACTCCCGGCTGAAA | 191.124.943 |
| 5p3 | CCAACCTCGGGAAAGTGACAGGCTG | AGGCTCTCGTGAGGTGGCTGGAATG | 194.944 |
| 5q3 | TAAAGACAGGGCTCCGAGGGTTGGG | CCTTCTCCAGCCATAAACCGGTGGG | 178.835.382 |
| 6p943 | GGAGCATGACGAGGGTGGCCCTGGAA | TGACGGAGGAGGTAGCAGCGACCTGGA | 858.213 |
| 6q | GCGTCCACTTGCTGTGACTGTGCTG | TGGGGCACCATGACTGCGAGAAGAG | 170.079.572 |
| 7p | GCCGCTGCATAACAAAGCACCCCAA | ACATGACACTGAGCCCACCTGCCTG | 791.647 |
| 7q500 | ACGGCTGACCCGAGGACATGCCTCC | TTGCGCCCTGAGACCCACCCCTTCC | 158.304.069 |
| 8p668 | TGCCACCACGGGAAGGCCACCTTTG | TTCACGCCACTGACGCCGCAGCTCC | 839.131 |
| 8q170 | TCCAGCACCAGCCCCACTACCCCAA | TCCCCTCACCCCACCCTTAGGCAGC | 146.087.066 |
| 9pFISH187* | TTCCCCGAACAACCTCGCCCGCTCC | TTCCCCCCGCGCTCAGACTTGCCTC | 205.285 |
| 9q520 | CCCGTCCCCTCGGTTCACACGACCA | GGCCCGCTGCCCAGGTATGCACAAG | 137.788.282 |
| 10p390 | GTCGCACCCTCCCCTCTCTGTGCCA | GCCTGTTGTTCCACCCACCCTCCCA | 425.269 |
| 10qF3 | TCACTTGGCACTGAGCCCCGCAAACC | GCCCCAACTCTGCCTGCCCTGTCAAC | 135.261.618 |
| 11p155 | GTCCCCAGCCACGCAGTGAACCTCC | GCCTGCACAGCCATCCCCAGCCTAC | 201.191 |
| 11q800* | TCCACTGACACCCGCAGAAGGGGCA | AGCACCGGGACTTCCTCAAGGGCCA | 134.142.465 |
| 12pFISH186 | ACCTACCAGCCCCCACCCAGCTTCC | AGCAGCCCCATGTGCTTCCCTGCC | 200.117 |
| 12qFISH400 | TGAACTCCACCGAGCGGTCGGAGCA | GGAAGTGCGGGCGACCAGACAGCAG | 132.041.928 |
| 13q200 | TCCGTCACCCCGCATCCATCACCCC | TGCCCAGCCGCCCAGTGACTCAGAA | 113.892.069 |
| 14q | AAGCACAGCCCCATCCACGAGGAAG | GGCCCCATGAAATGATCCCGGACCA | 105.375.613 |
| 15p* | ACAGGACATGGGAACACGGAGGGGA | CCACGGGGGCAAGTGGGATTGACAA | 26.168.736 |
| 15q1200 | AGACCCAGAGGAAGCGGAGCCAGCG | TGCGGCCTCCACTGCCCTGCATTG | 98.557.766 |
| 16p | TCAGCAGCCTCCTTACAAGGTGCCG | GTTGAATCCCAGGCCCCAGCACTCA | 1.051.724 |
| 16q | AGCTCCCCCAAGAGACAAGGCAGCA | AGCCCACACCGGCAGGATCTGATGA | 88.602.960 |
| 17p* | AAGGGAGGGGCTGGAGTGGAAGTCA | TGTTTCAGGCAGGTCGGGGACAAGG | 387.232 |
| 17qFISH | CCAGAGCCCACAGCCCCCTGGAGAA | TGGGGTGCGGTGTGGCTCCGTAGA | 77.739.799 |
| 18pFISH2 | ACTCCCCACGCTGTCCCACGCCAA | GCCAGGGGCTGACCTTGACCGCTCA | 648.757 |
| 18q800 | TGTGCCCCGCCACACAGCATCTGG | AGCAGGCAGTGCCCACCTTCCCTGA | 75.199.430 |
| 19pFISH300 | GGGAAGCGGCTGGGAACGGCAGAA | AGGCAGGGGCAGCGGGAGGAAGA | 292.434 |
| 19qFISH2 | CCCGCTGTGGGTGGGACTGGGGAAA | GGCACGGAAATCTCGGAGGGGGCCA | 63.723.434 |
| 20p291 | AAACAGCCCCATCGCCCCTCGACG | GGCACCCGCGCCACTCTTCCTGA | 275.962 |
| 20q | GGAAGGGCAAAGGCAAAGGGAAGGC | CACGGGGGCAGAGGCTAAACTCACA | 62.340.694 |
| 21q | TTCGTTCTGCTGTGGAAAGGCCCAG | CCCCAAAACCCGCATCGTGAGTTC | 46.905.828 |
| 22q | TCCAGGTGCCAGGAACTTCGGTCAG | AAGGATGGCCTTGGACCCTGTCAGG | 49.174.201 |
