# Supplementary material for: Identification of subtelomeric genomic imbalances and breakpoint mapping with quantitative PCR in 296 individuals with congenital defects and/or mental retardation
Source: Mol Cytogenet. 2009 Mar 12;2:10. doi: 10.1186/1755-8166-2-10 (PMC2660352; doi:10.1186/1755-8166-2-10)
Supplement: Additional file 2 — Supplementary table 2: Primers used for breakpoint characterization. Details for primers used for breakpoint charakterization. [file 1755-8166-2-10-S2.doc]

## Supplementary table 2: Primers used for breakpoint characterization

| **Name** | **Primer sequence 5’→3’** | | **Position** |
| --- | --- | --- | --- |
|  | *Forward* | *Reverse* |  |
| **1p** | | | |
| 01p008 | TGCAGTTCCTCCGCTGGCCTCCACG | GGGTTTGCAGCTCTGCGCCTGGCAC | 888.104 |
| 01pF | CCCCACCCCATGAGCTGGCACCAGA | CACGCTGCCACCTCGCCTGTCCATC | 1.030.272 |
| 01p215 | AGACCGTACTGGAAGGCGAGACCAT | ATGCCCATGACTTTGAGGATCTCCAG | 2.150.370 |
| 01p3.09 | AATATGTATGAGCCCAACCGGGACCT | GAAGTCTGCTGGGATCGGAATGTCTT | 3.092.569 |
| 01p467 | GGTCTCCATCCTACAAACACGGAAGA | AAGGACTCGGTGAACCCCTTAGTCTG | 4.672.405 |
| 01p626 | GCAATAAGTCCATGGAGATCGAGGTG | CTCAGCGACACGTAGGTGAAGAAGG | 6.263.786 |
| **1q** | | | |
| 01q240.1 | CCACCCACTTTGGGAACACTAAGAAG | CTCGCTCTTTAACTGCGACACATCAG | 240.108.954 |
| 01q241.6 | AACACTTGGAACAGGAGCAGCAGAAG | GTGAGGTGCAGTTGGTGCTCAGATT | 241.608.686 |
| 01q243.0 | CGACCAGATGGAGCTAGGAGAGGAG | AGCTCATCTTCCCCTTCCTGGAAAC | 243.093.799 |
| 01q244.0 | GGCTTCCCGATATCAACATCTACCAG | TCCGAGTACCATAGAACAAGGCTTCC | 244.088.437 |
| 01q2.2 | AGAGACACAGTGTGAAATCGCTGCTC | AGTGAGGACCTGAGCAGGTTCGATTA | 245.019.441 |
| 01q2.17 | GCTCGCCACAGCTTCCTTCACTAAAT | TTCCTTGGAACTTCTGACGCTGGAG | 245.079.548 |
| 01q1.59 | TTGTACGAGATGCAGGAGGAGGACTT | GACTCCACCCGATGACAGTTCTCAAT | 245.655.268 |
| 01q | TGGGTGCATGGGTGCTGACATGGTG | AGGCTCCAGGCTGCCTTGCAAACAC | 245.845.108 |
| 01q1.35 | ACCCTGGCCATAGGGGACTTTATTCT | GGGTCACCACTGTGAGATGAGATGAG | 245.902.228 |
| 01q1.14 | AGAGTGGGGTTTAGGGGTCTGTCAAG | CTTTCCAGTTGGAGAAGAGGCACTGA | 246.106.021 |
| 01q0.9 | TCGTCTCCACACACCGATGTACTTTC | GGTCAGGAAGAAGAAGCTTTGCACAC | 246.329.456 |
| 01q2 | AATCACAGCCCCACCCACACCTTCC | GCCATCTTGGGTACGGTGGTGCAGA | 246.433.047 |
| 01q3 | AGACAACCCTTCCTCCCCGACCTGA | TTCTGGGGGCTGCCAGCATTCTGA | 246.848.159 |
| 01q393 | GCTCATCCCCATCTCTATCATCTCCA | ACGTATGTGTAGAAGGCAGCCCCATA | 246.856.283 |
| 01q338 | ATCCAGTGTGATCCACCAGTTCTTCC | CTCGACTCTGTCCTGAAGGGATTCTG | 246.911.532 |
| 01q280 | GTAACGCCACCTGTCAGTTCCTGTCT | CCAAGAGGTAGAGGGAAGCTTTGGAG | 246.969.573 |
| 01q0.1 | CTGTACAACACCCTGAGCAGAAGCAC | CAATGGTTAGGGATCTGGGAGTGAGA | 247.074.409 |
| 01qF | CTTTCCCCCACCCCAGCAGCCACAC | GGAGGGAGGCAGAGAAGGGCAGGCA | 247.116.154 |
| **2p** | | | |
| 02pF | AGGAGGGGTCACCCTCACGCAGTGC | CATCCCTGCCTCAGCCTGTCCCCCA | 151.502 |
| 02p0.253 | CTGAGTTGGAAAGTGACACGAAGCTG | GCCTCGAGACCTGTTCTCTCAACTGT | 253.067 |
| 02p0.255 | CACCCTGAGAGGAAGGCCTAAATTGT | GTGAAGTGCTACCGAACAACGAAAGG | 255.712 |
| 02p2 | AACACCGCGAGGGACGTGCTAGAAG | CTGCAAAGTGGACAGCCGTGCAGTG | 306.513 |
| 02p963 | AGGGAGCAGCGTGAATGGTGGCAGGA | CAGGGGAAGGTGAGGCAGAGGACTGGG | 963.035 |
| 02p402 | CCACAGGGGGGAGCGTCAGGGATCA | CACGGCTGAGCGAGGACAGGGAGGA | 2.767.252 |
| **4p** | | | |
| 04pF | CGGCGTTTTCCTCTGGCTCCTGCGA | GCCCCGCACACTCACCATTTCCCGA | 43.211 |
| 04pF2 | CGCGAATGCTGTCCAAGCACTGCGTGA | GCAGACCCCAGGAGCCGCCTGTGAA | 320.044 |
| 04p | TGTCCAGCGACTCATCGCACGTCTC | AGAAAGCCGACAGCCGCAGAAGGAA | 739.120 |
| WHS1 1.85 | TAGAGCAGGGAGCAGAGAGCAGAACGGGGA | TGATACTCACCCCCACCCTCATGGAGCCAA | 1.853.368 |
| WHS2 1.98 | CCCTCTAGGTCATCACCACACCAGCAGCCA | TTCTCCAGCTTCCAGGAGCCGTCGTAGCA | 1.989.205 |
| **6p** | | | |
| 06pF | GGGCCTGGACAGAGCCAACTGGGGA | GAGACACAGGGTGGGTGGGGGTGGA | 171.059 |
| 06pIRF | GAAAGTTCCGAGAAGGCATCGACAAG | CTCCGCTCAACCAGTTCCTCAAAGTC | 339.840 |
| 06p943 | GGAGCATGACGAGGGTGGCCCTGGAA | TGACGGAGGAGGTAGCAGCGACCTGGA | 858.213 |
| 06p | AGCTCCTTGAACCCGGCTCAGGCTA | AGAAGCCCTGCCAGTGTCCCCTGAA | 880.018 |
| 06p1.3 | CGGCCAAGGCACCCAGGTCTCAGGAAA | ACTCCTACCCCCATCCGCTCCCCATCA | 1.355.617 |
| 06p1.8 | ACGACAGGCTCCACCCACAGCAGACA | TCTCCTCCTGCCTCTCCCTGCCAGTGA | 1.797.095 |
| 06p204 | ACTAAACACGTGCACCCAGGTT | TCAGGCAAGGACAACTACACCA | 2.040.287 |
| **6q** | | | |
| 6q165.71 | GGGCACAATATCTTCTCCACTCTGAGC | CGGTCTGGTACATCTCTTCCAACTGC | 165.712.704 |
| 6q166.76 | TGTGTACCTGGTAATGGAGCTGATGC | AATGGAGGTAGTCCATGGTCTTGGTG | 166.763.939 |
| 6q168.04 | CCATCACTGTGACCTTACCAACATGG | GATCCTGACTGGGGTCCACAAACTTA | 168.041.757 |
| 6q169.39 | CAGATGACCTCAGCAAGATCACCAAG | AATCCAGTAGGTGAGATCCAGCGTGT | 169.390.656 |
| 6q | GCGTCCACTTGCTGTGACTGTGCTG | TGGGGCACCATGACTGCGAGAAGAG | 170.079.572 |
| 6qF | CACAGCACCAGCCCCCTCCCCATCA | TGCCCATCGTGAGGAGCAGGTGCCA | 170.689.569 |
| 6q170.74 | TGCCTCAGCTCCTAAACTACCTGAA | GTAGACACTGTGTAAGCAATCTGTCAA | 170.728.435 |
| 6q170.75 | CGGACGCCTTCCACCGCTGCATCTT | GTCCCCCTGGTCGCTCCCGGAATTA | 170.735.232 |
| 6q170.8 | GGAAAAGACCAAACAGAAGGTGAGA | AGAAGAGGGTGACACACAGTTAGAA | 170.804.441 |
| **7p** | | | |
| 07pF | TGTGGTGGGATGGGTGATGGGGCGA | AGGGTGGTCTGAGGGCTGTGCTGGG | 138.955 |
| 07p152 | GAGCCAGGCCCAACACACAGGCCA | CCGGGAAGACCAGGAGGCACAGGGA | 152.277 |
| 07p188 | ACCGAAATTCACACTCAGGAGAGGAG | GAGGAGAAATAGCCGCTGGACTAGGT | 188.836 |
| 07p253 | GGCAATAGTGAGTGAGAGCTGGACAA | CGCTGTTTCAGAAGAGTGGCTGACTA | 253.453 |
| 07p290 | GGTGCTGAAGGTGCAGAATTCCTCT | CTGGAGTACAGCTCGTACCGGTTGAT | 290.647 |
| 07p686 | TCCAGTCAGGACTCGCTGTACCAATA | GAAAAGCCTTGCTGAGACCAGAGAAC | 686.796 |
| 07p711 | ATAAACCTTAGCGAGTGCGGCTGGA | GCCTTGAGGTTCCAGAGAAGCATCC | 711.619 |
| 07p | GCCGCTGCATAACAAAGCACCCCAA | ACATGACACTGAGCCCACCTGCCTG | 791.647 |
| **9p** | | | |
| 09pF187 | TTCCCCGAACAACCTCGCCCGCTCC | TTCCCCCCGCGCTCAGACTTGCCTC | 205.285 |
| 09pF553 | TGGCATGGGAAGGGGATGAGGCCGA | GGCAAATCCCGGTGGAACCCCGACC | 340.993 |
| 09p | GCAGGATTGGCACCCACACCCCTAA | TGGGGCTATGAGTGGCAACCCCAAC | 3.648.723 |
| 09p410 | CAAAGGGGAGGACTTCACTTGCTTCT | CCAGAGTGGACTCTCATGTGGATCAG | 4.107.790 |
| 09p507 | GAGATGTGCCGGTATGACCCTCTAC | CAGCACTGTAGCACACTCCCTTGTACT | 5.079.692 |
| 09p591 | CCTTCCACAGGTAGAAATGCTCTTGC | AGGCTGCACCTGAATCTTTACAGAGC | 5.912.729 |
| 09p6.24 | GTGCAGAGTGGGGATTGATGGTCTAT | GACCCCTGATATACCAAAGGCAAAGC | 6.241.091 |
| 09p6.4 | CATTTTCGTTCCCTCGGGTCTGTAAC | TTATAGAGCTGGAGCTCCCACATGGA | 6.403.077 |
| 09p6.52 | ACATAGGGATGCTTAACAGGGGTGGT | GCTGTGAGATGTTCTGGGAGGTTTCT | 6.532.185 |
| 09p7.16 | GCTCGATTTGTAAGTGCTGGCAGATG | CCAAGTATGTTTCCTGCTGGGCTTG | 7.159.882 |
| 09p7.79 | GGAGGAGAACTTCATTTCCCAGCAG | ATAGGACTCAAAAGGCTGGGACAACC | 7.789.699 |
| 09p8.3 | GCACTAGAGTACCTGGGCAGCTTTGA | CTCAAGTGCCCTGTATGGCTCAGAA | 8.307.792 |
| 09p8.99 | AGGAAGCGTTGGTACATTGTGGTGAC | TTACTGGGCTTCCGCCTACTTCTTTG | 8.992.688 |
| 09p10.6 | TCCCCATCACTATAGCCACAGAGGAC | TCCCCCAGCTATTAACACCCACTACC | 10.603.365 |
| **10q** | | | |
| 10q134.66 | TGGCCTGTGTGGACCGCTGTGGGGA | GGAGCCCAGCCCGAAGGGGAACCGAA | 135.079.396 |
| 10q20 | GTTGGTCGCAGGTGCCAGGCAGGA | GCAGCAGCCGAGGTGAGCACTGGAA | 135.104.878 |
| 10q134.73 | CCCTCTGTGCCTTTCCCTCCCCAGCC | GACTTCCAGCCCCTGTCCCAGAGCCC | 135.144.525 |
| 10qF2QIA | ACCGGGAAGGGGGAAGAGACCCACTGA | AGACGAGCCAGACCTCCAGACCGCAAC | 135.192.631 |
| 10q134.82 | TCGTGCCCTCCAACCTGCCCCATGAA | CCCCTCAGTGACCCCTGCCAAAGGGAA | 135.239.568 |
| 10q | TCTCCTTTGTGCCCGTGATGGTCCC | CTGCCACAACCAGTCGCCAAGCAA | 135.258.879 |
| 10qF3 | TCACTTGGCACTGAGCCCCGCAAACC | GCCCCAACTCTGCCTGCCCTGTCAAC | 135.261.618 |
| 10q134.87 | GACACCCCCACCCAGCCATTGTGCC | GCCCCTTCCCCTCGCATCTCGCAGA | 135.293.256 |
| 10q134.91 | GACCAGCCCCGCTGCTAACAGCTTCC | TGGCCCAGTGAGCATGGGGTGAACACA | 135.321.915 |
| **13q** | | | |
| 13q14.11 | GTGGGGGATGAGGGGCTGCTGAGGA | CGGGCCAGGCACTGAGGGGACATGA | 48.063.001 |
| 13q21.31 | CCACCAGGGCTTAGGGCAAGGAAGCCA | CCCCATGCCTCTCCCCTGCTGAGCAA | 69.385.361 |
| 13q31.3 | CGGCACCAACCAGGGAACACGCAACA | GGCTTGCAGCTTGCCAGGGAGTGTCC | 90.769.402 |
| 13q100.9 | AAAAGTCAGCTGAGTGCCGTACCTTG | AAGCAAACAGGTATCACCCACCAGAG | 100.903.859 |
| 13q102.3 | GTGGGCTTGTGGAAAGAGTCCTGTAA | AATTAGGGCTCCACAGTCAGGGATTC | 102.297.742 |
| 13q104.9 | GGTGCTGATTCTCTCCAGCTTTTCAG | AACCCTGACATGTTCCACTTCCTGTC | 104.916.657 |
| 13q105.9 | CCAGTGACATTATCATCCCGCTAAGG | GACATTAGGTGTCCTCTGGGAAAGCA | 105.943.342 |
| 13q106.6 | CAGTGTCATCAGCAACAAGACTGTGC | GTGGTGACACTCGTCCTTCCGTTACT | 106.620.825 |
| 13q107.3 | TCGAGGCTTACCAGGACTATGACCAC | ACCCCAACCAAGCAGAGATAGGAAAG | 107.315.971 |
| 13q108.1 | ACAGTGCTCCTTCTCACGCTTCAGTT | ACTAGTGAGCTGCCTCTGGAACAGGA | 108.118.995 |
| 13q109.6 | AATGAGAGTCACAGACTGGGGGTGAT | GCCTGGGAAGTGGCATTTGTAGAGT | 109.599.093 |
| 13q110.7 | CTGCAGACCTGCCTTAATGCTCAGAT | CCGAGGAAGATACTGGATCCACAGAA | 110.755.425 |
| 13q111.7 | GTTCTGTTAACTCACCGGGACCTTGA | TCTTGAGCCAAGACCTAGATGCCAAC | 111.773.354 |
| 13q112.6 | TGCACAGCAGAGAGACGTATGAGCTT | CACGTGTGTTTACACGTCTGGGAACT | 112.587.618 |
| 13q112.8 | ATGTTTGAGGCTGTAGCTCCCAGGAT | TTTAGTGATGCGTGTGTCCCTGACTC | 112.822.484 |
| 13q113.5 | CGCCCAGACCTTCTCCTTATCTTGTT | AGTAAAAAGTCACTGGGCCTGGGAAG | 113.550.272 |
| 13q200 | TCCGTCACCCCGCATCCATCACCCC | TGCCCAGCCGCCCAGTGACTCAGAA | 113.892.069 |
| 13qFISH | GAAGCAAGGACGCCTTCGCCAGCCA | GCCTGCAACCCCACCCTGCTCACAA | 113.950.696 |
| **19q** | | | |
| 19q53.0 | CCCCAAAAGCCCACGACCCCCCCAA | CGGAGTGCCTAGTGCCTCCACCTGCAA | 53.008.421 |
| 19q54.8 | CCCTCCACTCCCTCCCCATTCCTCCCA | CCACCCCACCCCCCCCAGCCATACATA | 54.820.858 |
| 19q56.9 | GAAGGTGGAGAAGGGCCAGGGCCAGA | ACCGCCCAACAGGCACAGAGCAGGA | 56.890.902 |
| 19q57.4 | GGGGCACCACCCCAGAGCCACAACA | ACCCTCTACCCCAAAGGCCAGCAGCA | 57.421.520 |
| 19q58.8 | AGGGAGCCCGAGCTACGGGGACAGA | ACGGAGCCAGATGATGGGGAGTGGGGA | 58.802.109 |
| 19q59 | CGCATCCCCTCAGCCTGTGGCACTCAA | ATCCTGCTACCCGCCCCCTCACCCAA | 58.982.725 |
| 19q59.7 | GCAAACACAGAGCCCACGGAAGGCCAA | TGAAGGCAGGGGCACTGGGAATGGGA | 59.746.408 |
| 19q60.35 | CCACCTACCCCGAAAGCCCCACCCA | AGGTCCCCTCCACCGTCGTCTCCGAA | 60.359.699 |
| 19q61 | GCCCAAGGACAGGCCCCCACCACAAA | GCTGTGTGAGGCATTGAGCCACCCGGA | 60.914.849 |
| 19q62 | ACCAGGGCAGCACCTGCACAGCAAA | TATGGGGCGGGGCAAGGCTGAAGTGA | 62.042.541 |
| 19q | GGCCAGGGACAGCCTGACAGAAACA | CAGGCCGAGAATCCCCTGCCTTGAA | 62.930.767 |
| 19qF2 | CCCGCTGTGGGTGGGACTGGGGAAA | GGCACGGAAATCTCGGAGGGGGCCA | 63.723.434 |
| 19qF250 | GCGCCGGAAGACGCCAGAGGAGCTA | ACAGCAACCTGCCCGACCACCCAC | 63.757.194 |
| **20q** | | | |
| 20q2 | GCAACAGCTTGGTGCCCGTGAACA | AACTCCAACAAGCAGCTCCAGGGCC | 44.315.799 |
| 20q5993 | ACAGACTCTGGAAACCCTCCCCTGT | CAGGATGAGGATGCAGATGAGGATG | 59.938.133 |
| 20q6099 | GAAGACAGGAGGTCCGAGGAGAAAG | CTTCTTTGGCACGAGGTTTCTAGGTG | 60.998.672 |
| 20qF2 | ATACCCCAGACCCCGCCCCACCAAC | TGGGCAGGAGGTGCAGCCCAGACA | 62.161.784 |
| 20q | GGAAGGGCAAAGGCAAAGGGAAGGC | CACGGGGGCAGAGGCTAAACTCACA | 62.340.694 |
